# Novel Use of PLGA Microspheres to Create an Animal Model of Glaucoma with Progressive Neuroretinal Degeneration

**DOI:** 10.3390/pharmaceutics13020237

**Published:** 2021-02-08

**Authors:** David Garcia-Herranz, Maria Jesus Rodrigo, Manuel Subias, Teresa Martinez-Rincon, Silvia Mendez-Martinez, Irene Bravo-Osuna, Aina Bonet, Jesus Ruberte, Julian Garcia-Feijoo, Luis Pablo, Elena Garcia-Martin, Rocío Herrero-Vanrell

**Affiliations:** 1 Innovation, Therapy and Pharmaceutical Development in Ophthalmology (InnOftal) Research Group, UCM, 28040 Madrid, Spain; davgar07@ucm.es (D.G.-H.); ibravo@ucm.es (I.B.-O.); jgarciafeijoo@hotmail.com (J.G.-F.); 2 Departamento de Farmacia Galénica y Tecnología Alimentaria, Facultad de Farmacia, Universidad Complutense de Madrid (UCM), IdISSC, 28040 Madrid, Spain; 3 Thematic Research Network in Ophthalmology (Oftared), Carlos III National Institute of Health, 28040 Madrid, Spain; mariajesusrodrigo@hotmail.es (M.J.R.); lpablo@unizar.es (L.P.); egmvivax@yahoo.com (E.G.-M.); 4 Department of Ophthalmology, Miguel Servet University Hospital, 50009 Zaragoza, Spain; manusubias@gmail.com (M.S.); teresamrincon@gmail.com (T.M.-R.); silviamendezmartinez@hotmail.com (S.M.-M.); 5 Miguel Servet Ophthalmology Research Group (GIMSO), Aragon Health Research Institute (IIS Aragon), 50009 Zaragoza, Spain; 6 Instituto Universitario de Farmacia Industrial (IUFI), Facultad de Farmacia, Universidad Complutense de Madrid (UCM), 28040 Madrid, Spain; 7 Center for Animal Biotechnology and Gene Therapy (CBATEG), Universitat Autònoma de Barcelona, 08193 Bellaterra, Spain; aina.bonet@uab.cat (A.B.); jesus.ruberte@uab.es (J.R.); 8 CIBER for Diabetes and Associated Metabolic Diseases (CIBERDEM), 28029 Madrid, Spain; 9 Department of Animal Health and Anatomy, School of Veterinary Medicine, Universitat Autònoma de Barcelona, 08193 Bellaterra, Spain; 10 Servicio de Oftalmología, Hospital Clínico San Carlos, 28040 Madrid, Spain; 11 Departamento de Inmunología, Oftalmología y ORL, Facultad de Medicina, Universidad Complutense de Madrid (UCM), IdISSC, 28040 Madrid, Spain

**Keywords:** PLGA microspheres, intraocular pressure, glaucoma animal model, intracameral injection, optic nerve, neurodegeneration

## Abstract

Progressive degeneration of neuroretinal tissue with maintained elevated intraocular pressure (IOP) to simulate chronic glaucoma was produced by intracameral injections of poly (lactic-co-glycolic) acid (PLGA) microspheres (Ms) in rat eyes. The right eye of 39 rats received different sizes of PLGA-Ms (2 µL suspension; 10% *w*/*v*): 14 with 38–20 µm Ms (Ms38/20 model) and 25 with 20–10 µm particles (Ms20/10 model). This novel glaucoma animal model was compared to the episcleral vein sclerosis (EPI) model (25 eyes). Injections were performed at baseline, two, four and six weeks. Clinical signs, IOP, retina and optic nerve thicknesses (using in vivo optical coherence tomography; OCT), and histological studies were performed. An IOP increment was observed in all three groups, however, the values obtained from the PLGA-Ms injection resulted lower with a better preservation of the ocular surface. In fact, the injection of Ms20/10 created a gentler, more progressive, and more sustained increase in IOP. This IOP alteration was correlated with a significant decrease in most OCT parameters and in histological ganglion-cell count for the three conditions throughout the eight-week follow-up. In all cases, progressive degeneration of the retina, retinal ganglion cells and optic nerve, simulating chronic glaucoma, was detected by OCT and corroborated by histological study. Results showed an alternative glaucoma model to the well-known episcleral vein model, which was simpler to perform, more reproducible and easier to monitor in vivo.

## 1. Introduction

Glaucoma is a degenerative optic neuropathy in which irreversible vision loss is produced by the gradual death of retinal ganglion cells (RGC). It has been estimated that this neurodegenerative disease will affect up to 111.8 million people by 2040 [1]. Increased intraocular pressure (IOP) is the universal risk factor and IOP fluctuations have been associated with immune-mediated progression of glaucoma [2,3,4]. However, the mechanism by which elevated IOP leads to RGC death remains unclear.

In order to broaden and improve knowledge of glaucomatous pathologies, several animal models have been developed in recent decades applying a significant increase in IOP secondary to a decrease in outflow of aqueous humor. To this, either laser, cauterization, ligature and/or episcleral vein sclerosis or mechanical blockage of the trabecular meshwork with obstructive substances (hyaluronic acid or paramagnetic, latex or polystyrene beads injected into the anterior chamber) have been used [5]. All these models produce a sudden, short increase in IOP resulting in limited RGC and axonal damage. Studies using non-biodegradable beads to create glaucoma animal models include a wide range of particle sizes, concentrations of administered suspensions and injection frequencies [6,7,8,9,10,11,12,13]. Although injections of these particles into the anterior chamber have been reported as causing RGC and optic nerve degeneration, undesired effects such as synechia and corneal opacity were also reported [14].

Although optic nerve degeneration and RGC apoptosis have been analyzed in glaucoma models using histological studies and computational technologies [15,16,17], discrepancies in retinal death distribution still remain. Furthermore, recent studies have even shown immune-mediated [18] contralateral eye degeneration [17].

The biodegradable aliphatic polyester co-polymer poly (lactic-co-glycolic) acid (PLGA) is approved by the U.S. Food and Drug Administration (FDA) and the European Medicines Agency for several clinical applications, mainly for drug delivery systems [19]. In an aqueous environment, PLGA degrades by hydrolysis, producing lactic and glycolic acid metabolized by Krebs’s cycle to CO_2_ and H_2_O being easily eliminated from the body [20].

In ophthalmic therapy, there is already an intraocular PLGA implant approved for clinical use (Ozurdex) [21]. This biodegradable polymer can also be used to produce microspheres (Ms). For several decades now, intraocular administration of Ms loaded with different active substances has been under evaluation as a therapeutic tool for diseases affecting the posterior segment of the eye [20,22]. The degradation rate of PLGA Ms ranges from weeks to months and is influenced by tunable factors such as polymer molecular weight, lactic:glycolic ratio, Ms size and shape, porosity, and pH environment [23,24].

This paper presents a new chronic animal glaucoma model created with repeated injections of biodegradable nonloaded PLGA Ms into the anterior chamber of rat eyes. This produces a progressive and chronic increase in IOP (up to eight weeks) and, consequently, a neurodegenerative process that simulates the conditions appearing in glaucoma patients. To our knowledge, nonloaded biodegradable microspheres have never been used for this purpose until now. The developed animal model shows a degeneration of photoreceptors and retinal ganglion cells that simulates human glaucoma.

## 2. Materials and Methods

### 2.1. Materials

Poly (d,l -lactide-co-glycolide) (PLGA) (50:50; inherent viscosity: 0.16–0.24 dL/g) was obtained from Evonik España, Granollers, Spain. Polyvinyl alcohol (PVA; 67,000 g/mol) (purchased from Merck KgaA, Darmstadt, Germany) and methylene chloride (obtained from Pan Reac Appli Chem, Barcelona, Spain) were also employed in microsphere manufacture.

### 2.2. Manufacture of PLGA Microspheres

The PLGA Ms were obtained using the oil-in-water (O/W) emulsion solvent extraction-evaporation technique. Thus, PLGA (400 mg) was first dissolved in methylene chloride (2 mL). Once dissolved, the organic polymer solution was emulsified with 5 mL of PVA Milli-Q water solution (1% *w*/*v*) using a homogenizer (Polytron RECO, Kinematica, GmbHT PT3000, Lucerne, Switzerland) at 7000 rpm for 1 min. The emulsion formed was then poured into 100 mL of PVA Milli-Q water solution (0.1% *w*/*v*) and magnetically stirred for 3 h to allow organic solvent evaporation and, subsequently, Ms maturation.

Afterwards, the Ms were washed with Milli-Q water to remove the PVA and were separated into two granulometric fractions (38–20 µm and 20–10 µm) using three sieves (mesh size 38, 20 and 10 µm). Finally, the Ms were freeze-dried (freezing: −60 °C/15 min; drying: −60 °C/12 h/0.1 mBar) and stored at −30 °C in dry conditions until use.

### 2.3. Microsphere Characterization

#### 2.3.1. Production Yield Percentage (PY%)

The production yield percentage was calculated using the following Equation (1):

PY% = (amount of microspheres)/(total amount of polymers) × 100(1)

#### 2.3.2. Morphological Evaluation

Morphological evaluation was performed on the freeze-dried Ms using scanning electron microscopy (SEM; Jeol, JSM-6335F, Tokyo, Japan). Gold sputter-coating was applied to the samples prior to observation.

#### 2.3.3. Mean Particle Size and Particle Size Distribution

Dual light scattering (Microtrac S3500 Series Particle Size Analyzer, Montgomeryville, PA, USA) was employed to determine mean particle size and particle size distribution. Three different measurements of each sample were obtained. The results were expressed as volume mean diameters (±standard deviation).

### 2.4. Animals

All work with animals was approved by the Ethics Committee for Animal Research (PI34/17, 27 June 2017) and was carried out in strict accordance with the ARVO Statement for the Use of Animals in Ophthalmic and Vision Research on the premises of the Biomedical Research Center of Aragon (CIBA). The animals were housed under controlled conditions (12-h dark/light cycle, temperature 22 °C, relative humidity 55%). Standard cages with environmental enrichment, water and food ad libitum were used.

Sixty-four Long Evans rats (40% males, 60% females) aged 4 weeks and weighing 50–100 g at the beginning of the experiment were used for this longitudinal and interventionist study. Long Evans rats were used as being easy handling and for comparison with previous studies having used these animals in glaucoma research and retinal degeneration [3,18,25,26,27].

### 2.5. Ocular Hypertension (OHT) Induction

All animals received a total of 4 ocular hypertension injections administered in the right eye (RE) at baseline, 2, 4 and 6 weeks under intraperitoneally sedative (60 mg/kg of Ketamine + 0.25 mg/kg of Dexmedetomidine), along with topical eye drops containing tetracaine 1 mg/mL + oxibuprocaine 4 mg/mL (Anestesico doble Colircusi, Alcon Cusí SA, Barcelona, Spain), under aseptic surgical conditions (iodized solution and erythromycin 5 mg/g ointment (Oftalmolosa Cusí eritromicina, Alcon Cusí SA, Barcelona, Spain)). Temperature was controlled using warm pads. Twenty-five animals received a sclerosing injection in the episcleral veins as previously described [28]. The other animals received PLGA Ms (2 µL suspension, 10% *w*/*v*) injected into the anterior chamber of the eye by superotemporal corneal puncture: 14 animals received Ms38/20 and 25 animals received Ms20/10. Injections were performed using a micrometered Hamilton syringe with a glass micropipette.

The injection was always done by an expert ophthalmologist using the same entrance door in the peripheral cornea, so the cornea was altered as little as possible. There were no central corneal leukomas that altered the corneal hysteresis.

### 2.6. Ophthalmological Studies

#### 2.6.1. In Vivo

Clinical signs such as ocular surface redness, corneal alterations, intraocular inflammation, infection or cataract formation, as well as IOP measurements using a Tonolab tonometer (Tonolab, TiolatOy Helsinki, Finland), were recorded every week. This procedure was accomplished using a sedative mixture of 3% sevoflurane gas and 1.5% oxygen for less than 3 min in order to avoid hypotension effects, as recommended by other authors [29]. The IOP value was obtained by averaging three consecutive measurements, taken from the average of 6 rebounds.

Neuroretinal structures were analyzed using optical coherence tomography (OCT Spectralis, Heidelberg Engineering, Heidelberg, Germany). Observations were performed at baseline and then biweekly up to week 8. A plane contact lens was adapted to the rat’s cornea to acquire high quality images. The protocols employed were as follows: retina posterior pole (R), retina nerve fiber layer (RNFL) and ganglion cell layer (GCL) with automatic segmentation. These protocols analyzed a circular area centered on the optic disc using 61 b-scans. Subsequent follow-up examinations were performed at this same location using the eye-tracking software and follow-up application. The retina and GCL were analyzed on the basis of the 9 ETDRS (Early Treatment Diabetic Retinopathy Study) areas [30], which included a central (C) 1-mm circle centered on the optic disc (though no fovea exists in rats) and inner (inferior; II; superior: IS; nasal: IN; temporal: IT) and outer (inferior: OI; superior: OS; nasal: ON; temporal: OT) rings measuring 2 and 3 mm in diameter, respectively, as well as total volume (TV). The RNFL protocol provided measurements of the 6 peripapillary sectors (inferotemporal: IT; inferonasal: IN; superotemporal: ST; superonasal: SN; nasal: N; and temporal: T). Retinal thickness composed from the inner limiting membrane to the retinal pigment epithelium; RNFL from the inner limiting membrane to the GCL boundaries; and GCL from the RNFL to the inner nuclear layer boundaries.

#### 2.6.2. Postmortem

Under general anesthesia, the animals were euthanized with an intracardiac injection of sodium thiopental (25 mg/mL). Enucleated eyes used for histology and immunohistochemistry were fixed in neutral buffered formalin (10%) and embedded in paraffin. A total of 18 REs belonging to 18 rats injected in the anterior chamber with Ms (20–10 µm and 38–20 µm) and injected with a saline solution in the episcleral veins (6 eyes each) were analyzed. As controls, the contralateral noninjected left eyes were used. Eyes embedded in paraffin were trimmed to reach the optic nerve head. After that, 5-µm sections were deparaffined, rehydrated and washed using H_2_O_2_ (10%) for 5 min (quenching) before incubation (overnight at 4 °C) of the following primary antibodies: mouse anti-Brn3a (Santa Cruz Biotechnology Inc., Dallas, TX, USA) at 1:50 dilution; and rabbit anti-glial fibrillary acidic protein (GFAP) (DAKO, Bath, United Kingdom) at 1:1000 dilution. Then, sections were incubated at room temperature (90 min) with specific secondary antibodies: biotinylated horse anti-mouse at 1:50 dilution and biotinylated goat anti-rabbit at 1:100 dilution (Vector Laboratories, Burlingame, CA, USA). After that, incubation was performed with ABC-HRP (Thermo Fisher Scientific, Waltham, MA, USA) at 1:50 dilution at room temperature (90 min). In a next step, sections were washed before and after every incubation in phosphate buffered saline solution finally, staining of sections was performed with diaminobenzidine (DAB) (3 min) and counterstained with Harrys Hematoxylin (Sigma-Aldrich Corp., St. Louis, MO, USA) at room temperature (20 min). Ganglion cells were counted in radial sections of the retina along 2 mm of a linear region of the ganglion cell layer, corresponding to four areas, two on each side of the optic nerve head. Images were then analyzed by an operator blinded to the treatment groups.

To analyze the location of the microspheres in the eye, paraffin sections stained with Hematoxylin/Eosin and with the fluorescent dye BODIPY (Invitrogen, Carlsbad, CA, USA) at 1:50 dilution were used. Once located, the nature of the cells surrounding the microspheres was analyzed by immunohistochemistry using rat anti-Mac2 (Cedarlane, Burlington, NC, USA) at 1:50 dilution. After incubation with biotynilated anti-rat antibody (Abcam, Cambridge, UK) at 1:250 dilution and Streptavidin Alexa Fluor 568 (Invitrogen, Carlsbad, CA, USA) at 1:100 dilution, nuclear counterstaining was performed with Bisbenzimide (Sigma-Aldrich, Madrid, Spain) at 1:100 dilution and microscopic analysis was performed using a laser-scanning confocal microscope (TCS SP2; Leica Microsystems GmbH, Heidelberg, Germany). Procedural immunohistochemistry controls were carried out by omitting the primary antibody in a sequential tissue section.

A total of 6 REs belonging to 6 rats injected in the anterior chamber with microspheres (20–10 µm and 38–20 µm) and injected in the episcleral veins (2 eyes each) with a saline solution were analyzed using transmission electron microscopy. As controls, the contralateral noninjected left eyes were used. Retinal fragments were fixed in 2.5% glutaraldehyde and 2% paraformaldehyde and then postfixed in 1% osmium tetroxide, stained in aqueous uranyl acetate, dehydrated and embedded in epoxy resin. Ultrathin sections (70 nm) were stained using lead citrate and examined using a transmission electron microscope (Jeol 1400, Jeol Ltd., Tokyo, Japan).

For scanning electron microscopy, a total of 4 REs were fixed in 2.5% glutaraldehyde and dissected. The iridocorneal angle was exposed to examine the presence of microspheres in the trabecular meshwork. After dehydration, samples were critical-point dried, mounted on stubs, coated with gold-palladium, and observed in a scanning electron microscope (JEOL JSM-5410, Tokyo, Japan) at an acceleration voltage of 20 kV using Semaphore software, Digital Micrograph v2.32, Gatan, Inc, Pleasanton, CA, USA (JEOL) for image acquisition.

### 2.7. Statistical Analysis

Data were recorded in an Excel database and statistical analysis was performed using SPSS software version 20.0 (SPSS Inc., Chicago, IL, USA). The Kolmogorov–Smirnov test was used to assess sample distribution. Nevertheless, due to the non-parametric distribution of most of the data, the Mann–Whitney U test was employed to evaluate the differences between both cohorts. A paired Wilcoxon test was also employed for change comparison recorded in each eye over the study period. Values were expressed as means (±standard deviations). Values of *p* < 0.05 were considered to indicate statistical significance. In order to avoid a high false-positive rate the Bonferroni correction for multiple comparisons was also calculated. The level of significance for each variable was established according to Bonferroni calculations (expressed as #). Statistical analysis of the number of ganglion cells was conducted on the GraphPad Prism 5 software (GraphPad Software, Inc., La Jolla, CA, USA) using one-way ANOVA. The results are shown as mean ± SEM. Values of *p* < 0.05 were considered to indicate statistical significance.

## 3. Results

### 3.1. Production Yield, Mean Particle Size and Particle Size Distribution

The PLGA Ms prepared showed a production yield (PY) of 44.94 ± 1.60% for the 38–20 µm size range, while the 20–10 µm fraction resulted in a PY of 39.37 ± 1.75%. In both cases, a monodisperse particle size distribution was observed in the selected size range) (see Section 3.2). The mean particle size for the 38–20 µm fraction and the 20–10 µm fraction resulted in 21.84 ± 1.26 µm and 14.07 ± 1.07 µm, respectively.

### 3.2. Morphological Evaluation

**Figure 1:**
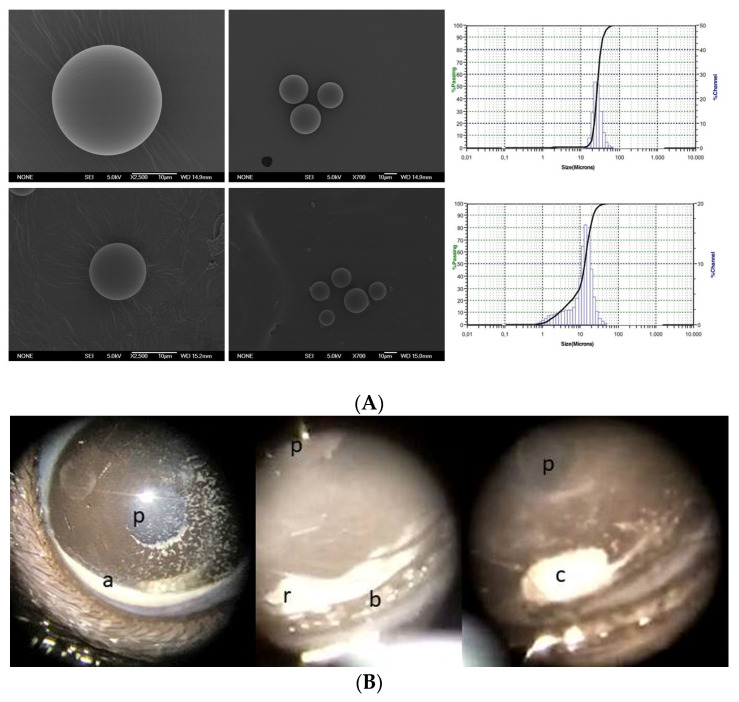
Visualization of microsphere (Ms) Scanning Electron Microscopy (SEM). (**A**) (SEM) images and poly (lactic-co-glycolic) acid (PLGA) microsphere particle size distribution, 38–20 µm Ms (top); 20–10 µm Ms (bottom) and monodisperse size distribution. Individual microspheres magnification 2500× and group microspheres 700× (**B**) Microsphere distribution in the anterior chamber of a rat eye during follow-up. Abbreviations: p: pupil; r: light reflex; a: microspheres at inferior iridocorneal angle; b: remnants of microspheres; c: microsphere pellet.

According to the SEM images, both size fractions contained spherical particles in the microrange with nonporous smooth surfaces (Figure 1A).

### 3.3. Ophthalmological Clinical Signs

No infection, intraocular inflammation (synechiae), cataract formation, or retinal detachment were found in any OHT model. It is worth mentioning that the corneal surface was better preserved in the Ms20/10 and Ms38/20 models than in the EPI model. As can be observed in Figure 1B, microparticles showed a tendency to aggregate and were localized at the inferior iridocorneal angle, sometimes forming a solid deposition (pellet). This arrangement allowed a clear visual axis and, subsequently, correct OCT acquisition.

### 3.4. Localization of Microspheres in Injected Eyes

**Figure 2:**
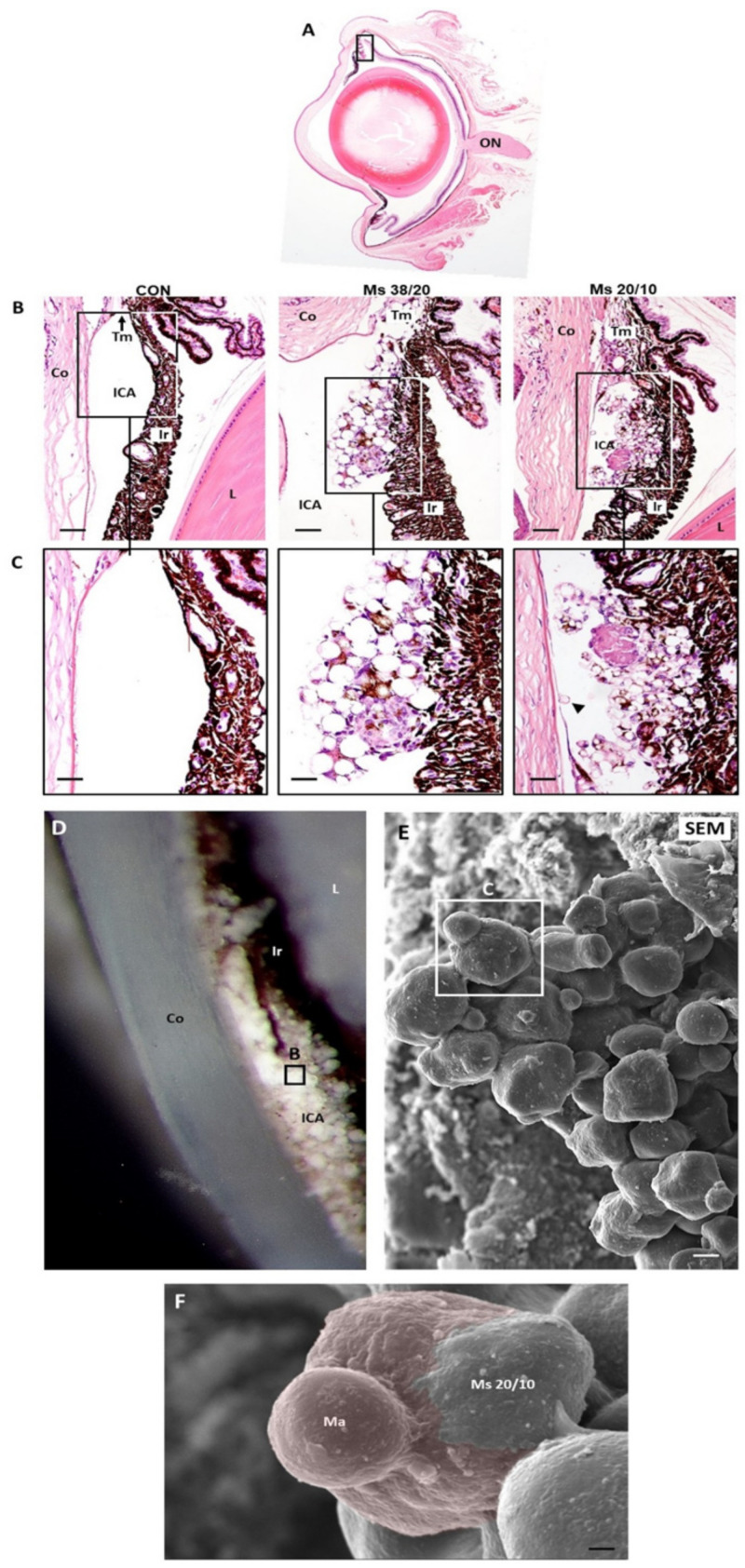
Visualization of microspheres at the iridocorneal angle with Hematoxilin/Eosin and scanning electron microscopy. (**A**–**C**) Localization of microspheres at the iridocorneal angle hampering the trabecular meshwork. Hematoxilin/Eosin histological sections at the level of the optic nerve. Arrowhead: microsphere in contact with the endothelium of the cornea. Scale bars for subfigure (**B**) is 53.47 μm and for subfigure (**C**) is 6.73 μm. (**D**–**F**) Dissection of the iridocorneal angle. The cornea has been sectioned and removed leaving the anterior eye chamber visible. Notice the whitish appearance of the microspheres. (**E**,**F**) Scanning electron microscopy images. In (**F**) an aggregate of microspheres is visible at high magnification. This PLGA structure is partially covered by two pseudopodia from a cell (colored in red) compatible with a macrophage. Scale bars for subfigure (**E**) is 5.4 μm and for subfigure (**F**) is 1.66 μm. Abbreviations: ICA: iridocorneal angle; ON: optic nerve; Tm: trabecular meshwork; Co: cornea; Ir: iris; L: lens; Ma: macrophage; CON: control eye; Ms38/20: eye injected with Ms size 38–20 µm; Ms20/10: eye injected with Ms size 20–10 µm.

**Figure 3:**
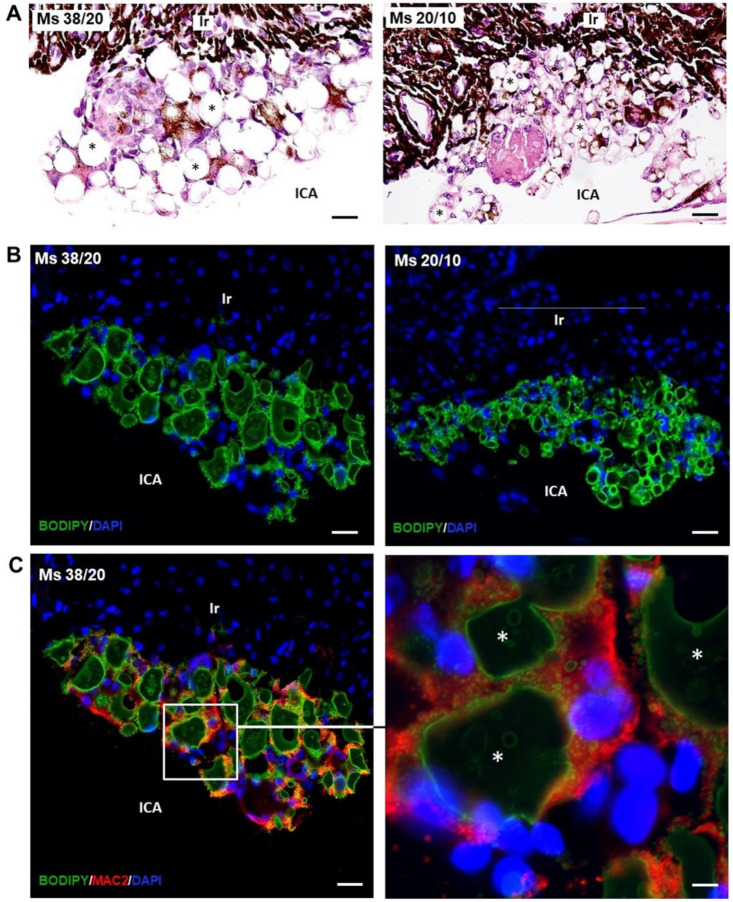
Identification of microspheres. (**A**) In Hematoxilin/Eosin-stained paraffin sections, microspheres (*) appeared transparent and colorless. They were surrounded by cells, sometimes containing black pigment. (**B**) Microspheres were specifically marked by boron-dipyrromethene (BODIPY) fluorescent dyes. (**C**) Double immunofluorescence using anti-Mac2 antibody, a specific marker of macrophages, showed that microspheres were surrounded by macrophages. Nuclei counterstained with Dapi. ICA: iridocorneal angle; Ir: iris; 38/20 and 20/10 µm microspheres. Scale bar: (**A**) 21.58 μm, (**B**) 18.49 μm, (**C**) 18.49 μm.

At eight weeks post-injection, a mixture of microspheres at different stages of biodegradation formed aggregates at the iridocorneal angle, hampering the trabecular meshwork (Figure 2). The microspheres were not colored in Hematoxilin/Eosin-stained sections, appearing as transparent structures (Figure 2C and Figure 3A). However, using the boron-dipyrromethene (BODIPY) fluorescent dye, PLGA microspheres were specifically marked in green (Figure 3B). Microsphere aggregates were stuck to the anterior surface of the iris (Figure 2C and Figure 3A). In contrast, only a few microspheres were in contact with the posterior epithelium (endothelium) of the cornea (Figure 2C). Pigmented and unpigmented cells appear inside aggregates surrounding the transparent microspheres (Figure 2C). These cells were marked with the anti-Mac2 antibody (Figure 3C), indicating that they belong to the macrophagic cellular lineage [31]. SEM images corroborate the presence of aggregates of microspheres and macrophages at the iridocorneal angle (Figure 2E,F).

### 3.5. Intraocular Pressure (IOP)

**Figure 4:**
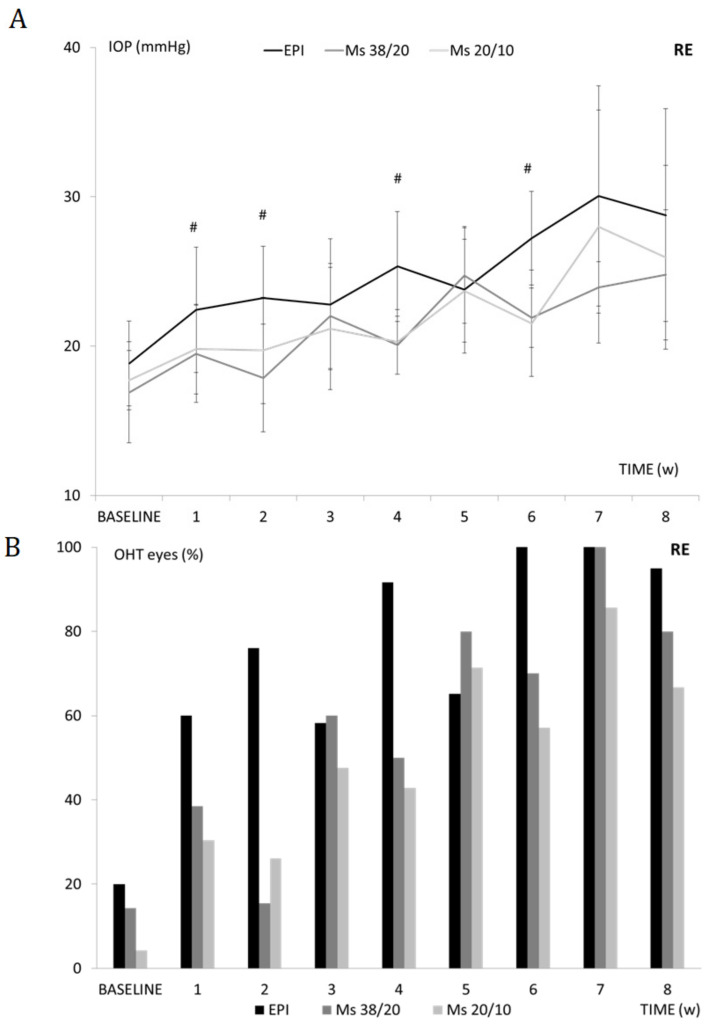
Analysis of intraocular pressure (IOP). (**A**) Right eye IOP curve in ocular hypertensive (OHT) models over follow-up. (**B**) Percentage of OHT right eyes (>20 mmHg) in the three OHT models over follow-up. Abbreviations: EPI: episcleral sclerosis model; Ms38/20: 38/20 microsphere model; Ms20/10: 20/10 microsphere model; RE: right eye; IOP: intraocular pressure; w: weeks, OHT: ocular hypertension; (%): percentage; #: statistical significance *p* < 0.020, for Bonferroni correction for multiple comparisons.

No differences were found among cohorts at baseline in terms of IOP values. Repeated injection of PLGA Ms into the anterior chamber produced continuous elevation of IOP. While OHT was detected three weeks after the first injection in both microsphere models, it was found from the first week onwards in the EPI model. The Ms38/20 model showed more fluctuations in IOP values than the Ms20/10 model (Figure 4A). High percentages of OHT eyes were found in all three models over time, although the EPI model showed the highest percentages at nearly all times examined (see Figure 4B). Left eyes also showed an increasing trend, albeit retarded (see Supplementary Materials: Figure S1).

### 3.6. Neuroretinal Examination

#### 3.6.1. In Vivo OCT

Ms38/20: REs showed a progressive decrease in retina, RNFL and GCL thickness. The thinnest sectors measured in the retina were the inner superior and inner temporal sectors; in the RNFL it was the nasal sector; and in the GCL they were the inner temporal and central sectors. Although a statistically decreasing trend was found in the retina and GCL, the retina and RNFL parameters showed an increase in thickness in the transition from weeks two to four (see Table S1). Left eyes (LEs) also experienced a statistical decrease in thickness in the retina, RNFL and GCL at week eight in most retinal sectors, as well as in the superotemporal sector of the RNFL and in the superior–inferior axis of the GCL (see Table S2).

Ms20/10: REs showed a decreasing trend in retina, RNFL and GCL thickness up to week eight. Although the retina protocol showed statistically significant decreased measurements in the central, outer inferior and temporal sectors, as well as in total volume, the inner superior retinal sector showed the lowest thickness at every examination. No statistical differences were found in the RNFL, although the superior nasal and nasal sectors exhibited the lowest thickness. Finally, the GCL showed significant statistical differences at week eight compared to baseline in the central and inner superior sectors. Interestingly, LEs showed even more OCT sectors with statistical decreases at week eight, while the thinnest sectors in both Ms models were the same (see Table S2).

In the episcleral model, the retina, RNFL and GCL experienced fluctuations in thickness throughout the study. Although the trend decreased in both eyes, no statistical differences were found at week eight.

##### Comparison of OHT Models

The OHT models were compared. REs did not show any statistical differences in retina and RNFL thicknesses at week eight; statistical differences were only found between the EPI and Ms20/10 models in the central (18.92 ± 3.47 vs. 15.68 ± 2.23 μm, *p* = 0.006#), inner inferior (26.25 ± 2.05 vs. 22.21 ± 4.32 μm, *p* = 0.009#) and inner temporal (23.50 ± 4.70 vs. 19.68 ± 3.74 μm, *p* = 0.047) sectors of the GCL.

**Figure 5:**
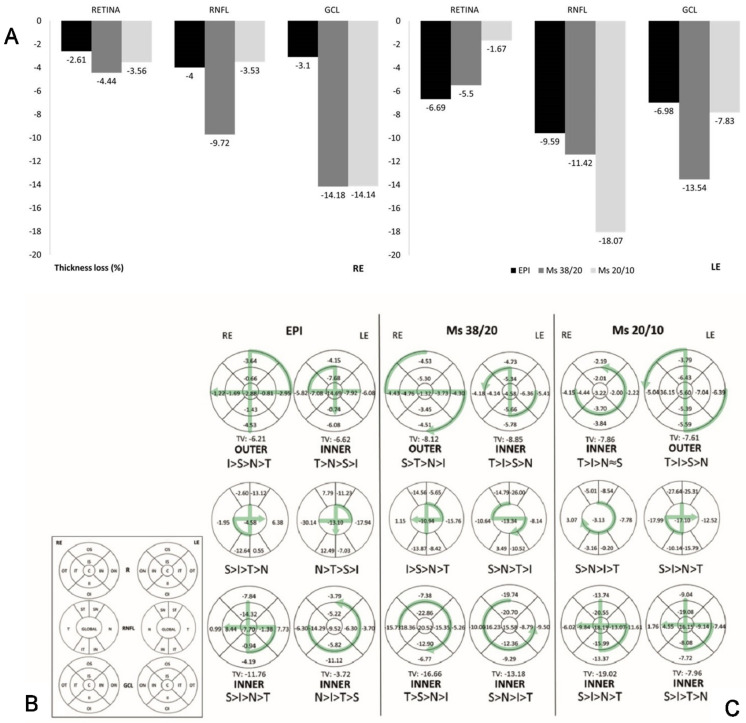
Percentage loss of neuroretinal structure identified by optical coherence tomography (OCT). (**A**) Percentage loss of retina, retinal nerve fiber and ganglion cell layers in the three ocular hypertensive models; (**B**) nomenclature of the neuroretinal sectors using OCT. (**C**) Neuroretinal percentage loss in OCT sectors and loss trend at 8-week follow-up. Abbreviations: R: retina; C: central; II: inner inferior; OI: outer inferior; IS: inner superior; OS: outer superior; IN: inner nasal; ON: outer nasal; IT: inner temporal; OT: outer temporal; RNFL: retina nerve fiber layer; IT: inferior temporal; IN: inferior nasal; ST: superior temporal; SN: superior nasal; N: nasal; T: temporal; GCL: ganglion cell layer complex; RE: right eye; LE: left eye; EPI: episcleral sclerosis model; Ms38/20: 38/20 microsphere model; Ms20/10: 20/10 microsphere model; TV: total volume; I: inferior; S: superior; N: nasal, T: temporal; >: higher loss than; (%): percentage; OCT: optical coherence tomography.

The perceptual loss of thickness detected by OCT (change in thickness with respect to the baseline measurement of each variable) was also analyzed. At the end of the study, the Ms38/20 model showed the highest percentage thickness loss in all OCT parameters in REs and in GCL in LEs. The GCL parameter experienced the greatest loss in each Ms model. Both the EPI and Ms20/10 models experienced this RNFL > GCL > retina loss trend in LEs. Nevertheless, no statistical differences in thickness were found between REs and LEs in the Ms models (Figure 5A).

REs from the EPI and Ms38/20 models showed greater loss in the outer retinal sectors. An I > S > N > T loss trend was found in the EPI model. Nevertheless, the retinal temporal sector experienced the greatest percentage loss in both Ms models and in LEs in the EPI model. In all models, both eyes showed greater percentage loss in the superior–inferior axis sectors of the RNFL. The exception was the LE in the EPI model, which experienced the inverse of the RE loss trend. Moreover, in all OHT models both eyes showed greater loss in the inner sectors of the GCL, and REs in both the EPI and Ms20/10 models experienced the same percentage loss trend by OCT sector (S > I > N > T) (see Figure 5B,C).

The RE loss rate per day and IOP increase in mmHg per week were calculated from the average of all OCT sectors and expressed in μm/mmHg/day. The Ms38/20 model experienced the highest loss rate (on average) in the retina, RNFL and GCL over the study and at most examinations. The Ms20/10 model experienced a gentle, progressive and sustained decrease from weeks two to eight. This did not occur with the EPI model, which in the earliest examinations (weeks two and four) even showed an increase in the RNFL and GCL. The EPI and Ms20/10 models experienced similar loss rates in RNFL (0.0033 vs. 0.0030 μm/mmHg/day) at the end of the study (week eight) (see Table S3).

**Figure 6:**
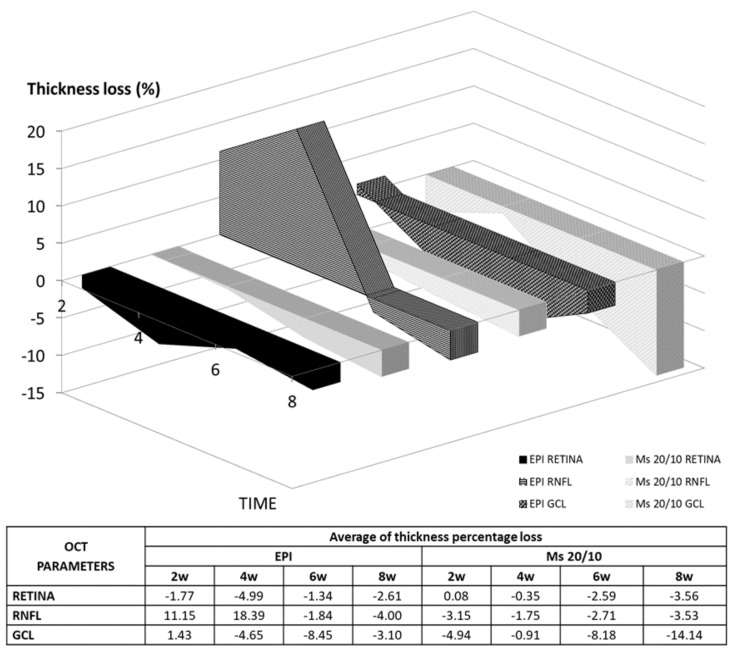
Changes in thickness percentage loss measured by optical coherence tomography (OCT) in two ocular hypertensive (OHT) models. Abbreviations: EPI: episcleral sclerosis model; Ms20/10: 20/10 microsphere model; RNFL: retina nerve fiber layer; GCL: ganglion cell layer complex; w: week; %: percentage.

As comparable results were found between the EPI and Ms20/10 models, deeper analysis was performed to determine which model was more aggressive or induced neuroretinal damage earlier. Statistical differences were only found in 12 of the 135 parameters, and in 10 of them the Ms20/10 model showed lower thickness up to week eight (see Table S4). Figure 6 shows the neuroretinal changes in the REs based on average percentage of thickness loss up to week eight. The EPI model had the most intense and rapid loss in RNFL thickness from weeks four to six. Although the Ms20/10 model showed a progressive decrease, it produced the greatest percentage loss in GCL later on.

#### 3.6.2. Postmortem Examination

**Figure 7:**
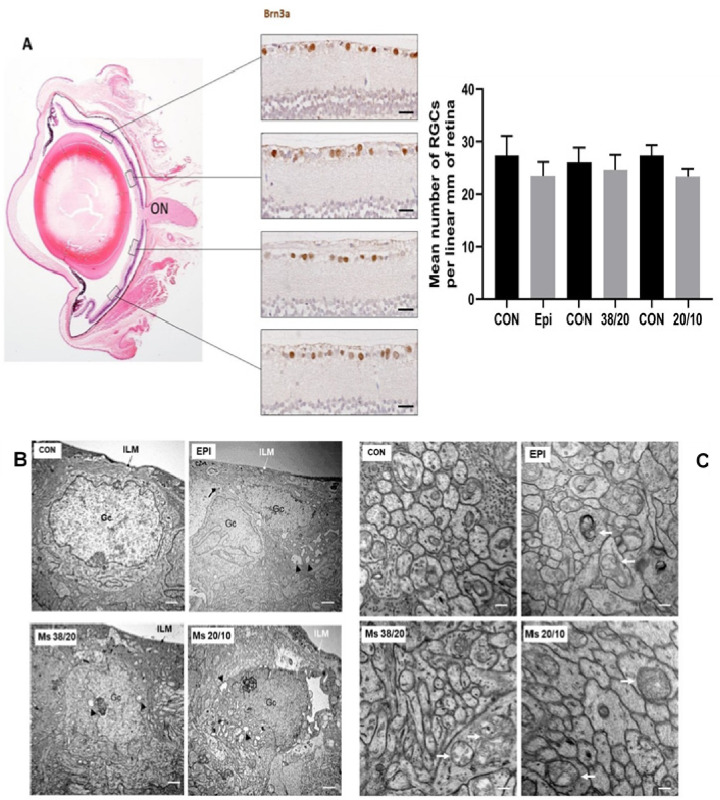
Neurodegeneration of (**A**) ganglion cell layer, (**B**) somas and (**C**) axons. (A) Ganglion cell analysis. Ganglion cells were counted in radial sections of the eye along 2 mm of a linear region of the retina. These corresponded to four areas, two on each side of the optic nerve (ON). Nuclei of ganglion cells were marked with anti-Brn3a. Right image: mean number of ganglion cells per linear mm of retina. Scale bars: 27.73 μm. (**B**) Degeneration of ganglion cells in OHT models at 8 weeks. Nuclear and nucleolar ultrastructural alterations, including indentations of the nuclear membrane and cytoplasmic vacuolization were observed in all OHT models. Scale bars: 1.13 μm. (**C**) Enlarged mitochondria (arrows) were observed in the axons of ganglion cells in all OHT models at 8 weeks. Scale bars: 0.29 μm. Abbreviations: EPI: right eyes injected in the episcleral vein; Ms38/20: right eyes injected with 38–20 µm microspheres; Ms20/10: right eyes injected with 20–10 µm microspheres; RGC: ganglion cell; CON: right eye from a nontreated rat; GC: ganglion cell; ILM: internal limiting membrane.

**Figure 8:**
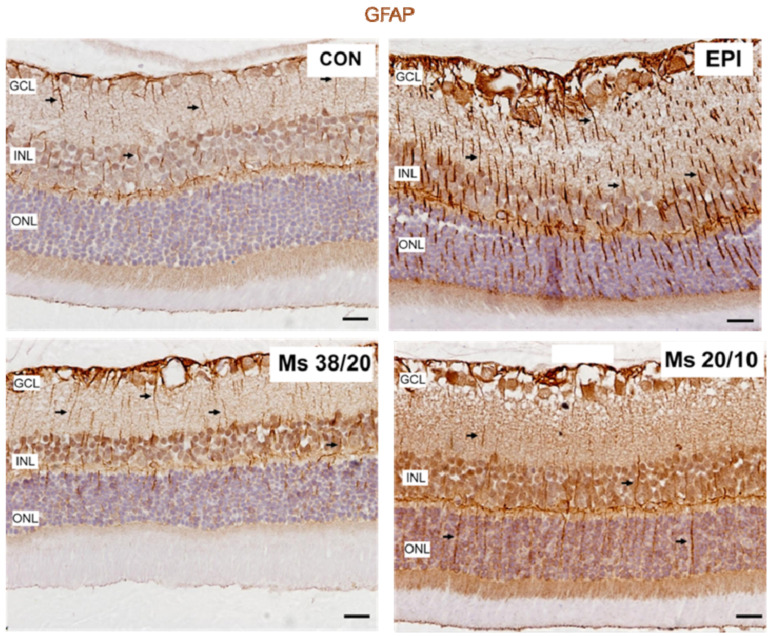
Overexpression of GFAP (gliosis) was observed in retinas of all ocular hypertensive (OHT) right eyes at 8 weeks. Note the expression of GFAP in the cytoplasmatic prolongations of Müller cells (arrows). The retinas of contralateral (left) eyes also showed gliosis, although the expression of GFAP by Müller cells was lower. Abbreviations: CON: representative retinal section from a contralateral (left) eye; EPI: representative retinal section from a right eye injected in the episcleral vein; Ms38/20: representative retinal section from a right eye injected with 38–20 µm microspheres; Ms20/10: representative retinal section from a right eye injected with 20–10 µm microspheres; GCL: ganglion cell layer; INL: inner nuclear layer; ONL: outer nuclear layer.

At eight weeks postinjection, retinal morphology was analyzed in the hypertensive rat models. No obvious histological signs of retinal degeneration were observed (Figure 7A). However, ultrastructural analysis showed a process of ganglion cell and photoreceptor degeneration (Figure 7B,C). Common lesions observed in ganglion cells in all the hypertensive models were indentations of the nuclear membrane and cytoplasmic vacuolization (Figure 7B). Furthermore, as occurs in experimental glaucomatous mice after injection of microbeads in the anterior chamber [32], the axonal mitochondria of ganglion cells in all hypertensive rat models were enlarged and degenerated, showing fewer cristae (Figure 7C). Brn3a-positive ganglion cells were counted in radial sections of the retina along 2 mm of a linear region of the ganglion cell layer. These corresponded to four areas, two on each side of the optic nerve head (Figure 7A) No significant differences in the number of ganglion cells were found among the hypertensive models (EPI 23.51 ± 2.59 vs. MSs38/20 24.50 ± 2.96 vs. MSs20/10 23 ± 1.15 mean number of ganglion cells per linear mm of retina, *n* = 6, *p* = 0.901) (Figure 7A). Micronuclei and loss of heterochromatin were observed in the rod photoreceptors of all hypertensive models. Increased expression of GFAP in cytoplasmic prolongations of Müller cells (gliosis) was observed in the entire retina of all hypertensive models (Figure 8).

## 4. Discussion

To our knowledge this is the first study focused on the intracameral injection of biodegradable PLGA Ms as a method to induce chronic ocular hypertension. In this work we analyzed two different Ms sizes (small: 20/10; and large: 38/20) and compared them with the well-established Morrison model (EPI) [28]. Although in many previous studies researchers injected beads into the anterior chamber, they all used non-biodegradable materials [5]. This is the case of [8] the glaucomatous effect comparison after injecting a suspension (2 microliters) of non-biodegradable polystyrene microparticles of different sizes and concentrations: 10 microns (106 beads/mL) and 15 microns (5 × 106 beads/mL) into the anterior chamber. In that study, the small microparticles (10 microns) produced a greater and more sustained increase in IOP than the large ones (15 microns), which only increased IOP for two weeks. In the present study, the repeated injection of the two particle size fractions tested (20–10 µm and 38–20 µm) produced an increase in IOP after Ms injection followed by a decrease at one week resulting in fluctuating IOP values. The larger Ms (Ms38/20) produced greater fluctuations and resulted in both lower final IOP and greater degenerative loss as evaluated by OCT at eight weeks. This suggests that fluctuations in IOP could play an important role as an accelerator of the disease [2].

In this study, a comparison with a cohort injected with the vehicle employed to create the particle suspensions (saline solution) was not performed as other authors have already demonstrated that axon density in the nerves injected with saline did not differ from the ones observed in naive nerves [6].

In order to compare results between the different models, the authors decided to carry out biweekly injections. According to the results obtained, reinjection of Ms resulted in the accumulation of particles in the administration site accompanied by progressive in situ degradation. This combination of factors probably produced obstructions in the trabecular meshwork, generating a gradual increase in IOP. It is worth mentioning that the injection of the smaller Ms avoided potential peaks and postinjection variability. Although these factors mainly occur after episcleral injections, they are also produced by injection of large Ms. According to the results observed, injection of Ms20/10 created a gentler, more progressive and more sustained increase in IOP that focused the harmful effect of OHT and allowed it to be isolated.

In the present work, after intracameral injection of PLGA Ms, a whitish material and aggregation of particles were observed in the aqueous humor. Both phenomena were similar to those described after intravitreal injection of PLGA particles [33,34]. Furthermore, it is important to remark that the repeated injections of Ms (0.2 mg/injection) increased the amount of PLGA at the administration site, resulting in a total of 0.8 mg by the end of the study (eight weeks), therefore with the particles in different stages of degradation. The remains of microspheres were observed in the biweekly reinjections and until the end of the study (eight weeks). The frequency of the injections made it impossible to know if the remains belonged to two, four, or six weeks, however the visualization of remnants of different sizes suggested a durability of at least more than four weeks. The different shapes found in the site of injections can be explained from the biodegradation process of the PLGA particles. In fact, it has been previously reported that PLGA microspheres’ morphology changes over time. For example, in a previous study, authors observed that microparticles prepared with the same polymer used in the present work were able to maintain their spherical shape up to two weeks after incubation in PBS and that from four weeks onwards the particles suffered erosion and shape loss [35], coinciding with our findings.

Our histological results also demonstrated macrophage affectation and activation by PLGA Ms (Figure 2F), this time inside the anterior chamber of the rat eye. Other authors [33] described a localized foreign body reaction partially surrounding the degraded PLGA microspheres after intravitreal injection. Histologic studies also demonstrated the presence of mononuclear cells and multinucleated giant cells with no involvement of the retina [36]. The reaction described by these authors is similar to the one observed after intramuscular injection of microspheres in rabbits, decreasing over time. It has been reported that macrophages have the ability to phagocytize PLGA particles with sizes below 10 µm [37] and that these effector cells at the interface of the biodegradable microspheres can produce acid and other agents and may decrease interfacial pH [34]. PLGA microspheres as ocular drug delivery carriers are intended to avoid frequent injections. Therapeutic use of microspheres also includes drug loading, different to the blank microspheres used in this work. In the present study, the amount of microspheres at the trabecular meshwork and the frequent injections caused an inflammatory response which was accompanied by macrophages at the surface of the microspheres (Figure 2). As described by other authors, macrophages at the interface of the biodegradable microspheres can produced acidic concentrations and low the pH of the extracellular fluids [37]. It should be noted that an acidic environment is also present in glaucoma, supporting the advantage of using nonloaded PLGA microspheres to create glaucoma animal models. It is important to remark that the behavior of nonloaded and loaded PLGA microspheres is different as the microenvironment pH created as a result of the injection of the loaded particles depends not only on the polymer but also the drug. In fact, it has been demonstrated that the use of basic drugs or additives can led to a pH increase and that appropriate amounts of basic substances can neutralize carboxylic end groups [38].

Evaluation using OCT revealed that the increase in IOP resulted in neuroretinal loss both in the inner sectors of the GCL and in the sectors of the superior–inferior axis of the RNFL, as several authors found [15,17,28], but contrary to the findings of other authors [8,18] that detected peripheral loss. These discrepancies between studies might be due to retinal growth of the eye at the expense of peripheral areas [39]. A rate of RNFL loss similar to that reported by other authors [15] was also found, occurring in both the episcleral model and in the Ms20/10 model, which supports the reproducibility of our results and the similarity between the models in this eight-week study.

Recent studies have demonstrated bilateral neurodegeneration following unilateral induction of hypertension using different models such as episcleral vein sclerosis, cauterization of episcleral veins and of the trabecular meshwork, or an intermittent loop [3,17,18,40]. It is generally accepted that glaucomatous damage is a consequence of axonal degeneration that leads to retinal ganglion cell death. Glial activation is also present in glaucoma [41]. The affectation of microglia in neurodegeneration and its progression have been demonstrated [18] in previous methods for inducing hypertension. All of them are aggressive and produce sudden increases in IOP, which suggest the presence of an immune reaction to a stressor. However, it has also been recently suggested that latent inflammation would worsen retinal degeneration [42], as we found in our results, which showed increased gliosis even in Ms models that produced gentle increases in IOP. The present work would be the first study demonstrating that a gentle and sustained increase in IOP also resulted in contralateral retinal damage, as shown by the neuroretinal decrease revealed by OCT and the fact that, despite the decrease in the number of RGC, no statistical differences were found between the eyes (contralateral eye and induced eye) (Figure 7A). This could be due to the use of the opposite eye as a control in our study. As we previously mentioned, several authors have recently demonstrated the involvement of the opposite eye after the induction of ocular hypertension [3,17,18,40], so it is recommended to take this aspect into account or to use healthy animals for comparison in future studies. Other authors have already shown that a small increase in IOP, relative to the contralateral eye, could trigger the T-cell-mediated neurodegenerative process [4]. This damage has been detected after 24 h in the induced eye [40] and after one week in the uninduced contralateral eye [17]. In this study, OCT showed neuroretinal loss from the first interim scan (two weeks), though it could possibly be detected even earlier. As glial activation is now known to spread via the visual pathway, degenerative factors could, in turn, activate the laminar and prelaminar glia [43] of the contralateral eye, producing retrograde damage and also creating an imbalance in pressure in the optic nerve head [44]. On the other hand, in addition to this damage to the neuroretinal structure, a gradual increase in IOP was also detected in the uninduced contralateral eye, as also found by other authors [18,45].

It is important to remark that injection of PLGA Ms in the anterior chamber is an easy surgical technique to generate OHT and, moreover, adequately maintains ocular structures, making this a simple, useful and efficient hypertensive model. In this study, the small volume of only 2 µL was chosen to avoid a significant increase in the initial intraocular pressure and to facilitate a more progressive mechanical blockage of the particles in the site of injection. In a previous work, it was shown that an injection volume of 2.5 mL of saline solution generated IOP fluctuations about a consistent baseline and also that volumes up to 7 µL could even be administered since the values of the mean IOP for the eyes injected with saline did not depend on injection volume [6].

In the current study, the importance of the administration route and the nature of the device in the observed effect has also been demonstrated. The intracameral route is used for the administration of antibiotics and recently several intracameral implants are undertaking clinical trials or are already commercialized [46]. In the case of implants and from a clinical point of view, one of the side effects of the intracameral implantation is related to the iridocorneal angle. In fact, the use of therapeutic devices such as the case of Durysta, an implant made from PLGA and bimatoprost (Allergan, Irvine, CA, USA), is restricted in patients with small angle or any issue that could promote the deposit of the device in the inferior angle [46]. Taking into account this mentioned side effect, we used the microspheres to promote an obstruction of the trabecular meshwork which in turn promotes an increase of IOP. Furthermore, the aggregation of the administered particles and their high frequency of injection (baseline, two, four and six weeks) allow the microspheres to remain longer in the site of injection. This produces a mechanical blockage that has been previously described in other studies by using non-biodegradable particles [6,7,8]. It is worth mentioning that in this case the injection of the microparticles is not used for therapeutic purposes and also that the aggregation at the iridocorneal angle is one of the causes of the increase of IOP. By using other administration routes, mainly intravitreal and periocular, PLGA microspheres have demonstrated their potential for the treatment of several ocular diseases due to the ability of these systems to deliver multiple active substances directly to the target site during the long term [20,22]. Previous studies have demonstrated that microspheres for therapeutic purposes were well tolerated after intravitreal injection [20,35,47,48]. The amount of PLGA microspheres is also an important factor to be considered. Other studies have shown that a high quantity of microspheres can induce retinal damage, being well tolerated if low amounts of these drug delivery systems are used [49]. In the present study microspheres were injected every two weeks resulting in a total of 0.8 mg (high quantity in rat trabecular meshwork) at the end of the study.

Based on the results obtained in the present work we could predict that future studies with longer follow-up times and less neurodegenerative damage (by modulation/minimization of reinjection) would allow getting closer to real-world glaucoma models. Chronic glaucoma animal models are of great interest for the study of the different phases and stages of glaucoma, and to test new treatments based on long-term drug delivery systems.

In conclusion, this study describes a new glaucoma model using biodegradable PLGA microspheres injected into the anterior chamber of the eye that causes a progressive increase in IOP and neuroretinal degeneration like the ones observed in the episcleral vein model. This study paves the way for new models of neuroretinal degeneration using a method that is simpler to perform and more reproducible than episcleral vein injection. This model is also less aggressive to the surface of the animal’s eye, thereby making transparent structures available in that eye. This fact allows repeated evaluation by noninvasive technology such as OCT to monitor the development of the pathology generated and to quantify in an objective and nonharmful way the effectiveness of new therapies to treat glaucoma.

## Data Availability

The data presented in this study are available on request from the corresponding author.

## Supplementary Materials

The following are available online at https://www.mdpi.com/1999-4923/13/2/237/s1, Figure S1: (A) Left eye IOP curve in the OHT models during follow-up. (B) Percentage of OHT left eyes (>20 mmHg) in the three OHT models during follow-up. EPI: episcleral sclerosis model; Ms38/20: 38/20 microsphere model; Ms20/10: 20/10 microsphere model; LE: left eye; IOP: intraocular pressure; w: weeks; OHT: ocular hypertension; (%): percentage; *: statistical significance *p* < 0.050; #: statistical significance *p* < 0.020 for Bonferroni correction for multiple comparisons, Table S1: Structural neuroretinal analysis using OCT with the Ms38/20 model. Ms38/20: 38/20 microsphere model; RNFL: retina nerve fiber layer; GCL: ganglion cell layer complex; thickness in microns (μm); mean ± SD (SD: standard deviation); *: *p* < 0.050 statistical significance; #: *p* < 0.020 statistical significance with Bonferroni correction for multiple comparisons. Grey cells show the two thinnest sectors at every examination, Table S2: Left eye neuroretinal analysis using OCT in both microsphere models at week 8. Ms38/20: 38/20 microsphere model, Ms20/10: 20/10 microsphere model; RNFL: retina nerve fiber layer; GCL: ganglion cell layer complex; thickness in microns (μm); mean ± SD (SD: standard deviation); *: *p* < 0.050 statistical significance; #: *p* < 0.020 statistical significance with Bonferroni correction for multiple comparisons; w: week. Grey cells show the two thinnest sectors at every examination, Table S3: Right eye neuroretinal loss rate measured using OCT in the OHT models. EPI: episcleral sclerosis model; Ms38/20: 38/20 microsphere model; Ms20/10: 20/10 microsphere model; RNFL: retina nerve fiber layer; GCL: ganglion cell layer complex; RE: right eye; thickness in microns (μm); w: week. Grey cells show the lowest measurements, Table S4: Right eye neuroretinal analysis follow-up using OCT in both OHT models. EPI: episcleral sclerosis model; Ms20/10: 20/10 microsphere model; RNFL: retina nerve fiber layer; GCL: ganglion cell layer complex; thickness in microns (μm); mean ± SD (SD: standard deviation); % Ch: percentage change in thickness loss; *: *p* < 0.050 statistical significance; #: *p* < 0.020 statistical significance with Bonferroni correction for multiple comparisons; w: week. Cells colored grey when EPI model showed thinner sectors and/or higher percentage loss compared to Ms20/10.
