# Supplementary material for: Novel Use of PLGA Microspheres to Create an Animal Model of Glaucoma with Progressive Neuroretinal Degeneration
Source: Pharmaceutics. 2021 Feb 8;13(2):237. doi: 10.3390/pharmaceutics13020237 (PMC7915113; doi:10.3390/pharmaceutics13020237)
Supplement: Supplementary file 1 [file pharmaceutics-13-00237-s001.pdf]

Supplementary Materials: Novel Use of PLGA Microspheres to Create an Animal Model of Glaucoma with Progressive Neuroretinal Degeneration

David Garcia-Herranz, Maria Jesus Rodrigo, Manuel Subias, Teresa Martinez-Rincon, Silvia Mendez-Martinez, Irene Bravo-Osuna, Aina Bonet, Jesus Ruberte, Julian Garcia-Feijoo, Luis Pablo, Elena Garcia-Martin and Rocío Herrero-Vanrell

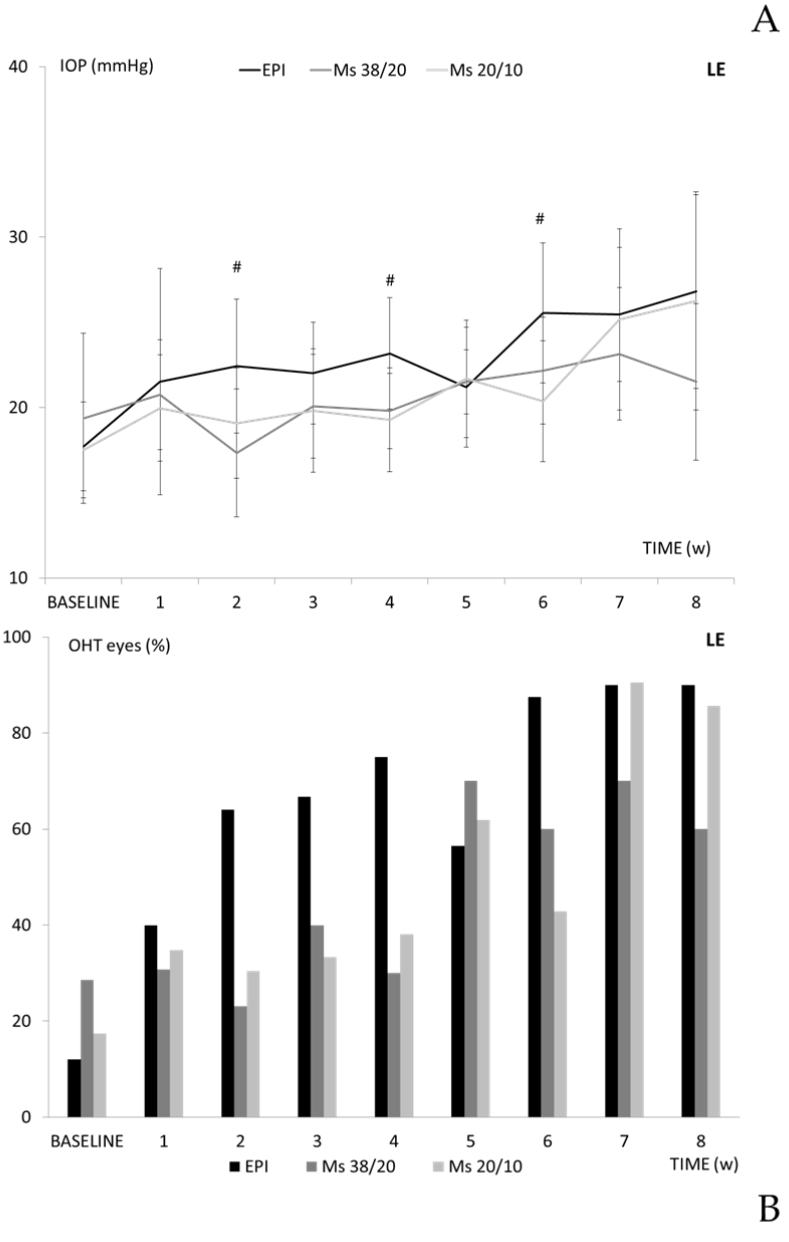

Figure S1. (A) Left eye IOP curve in the OHT models during follow-up. (**B**) Percentage of OHT left eyes (>20 mmHg) in the three OHT models during follow-up. EPI: episcleral sclerosis model; Ms38/20: 38/20 microsphere model; Ms20/10: 20/10 microsphere model; LE: left eye; IOP: intraocular pressure; w: weeks; OHT: ocular hypertension; (%): percentage; *: statistical significance $p < 0.050$; #: statistical significance $p < 0.020$ for Bonferroni correction for multiple comparisons.

Table S1. Structural neuroretinal analysis using OCT with the Ms38/20 model. Ms38/20: 38/20 microsphere model; RNFL: retina nerve fiber layer; GCL: ganglion cell layer complex; thickness in microns (μm); mean ± SD (SD: standard deviation); *: $p < 0.050$ statistical significance; #: $p < 0.020$ statistical significance with Bonferroni correction for multiple comparisons. Grey cells show the two thinnest sectors at every examination.

Right eye (Ms38/20)						
**OCT parameters (μm)**	**Baseline Mean ± SD**	**2 w Mean ± SD**	**4 w Mean ± SD**	**6 w Mean ± SD**	**8 w Mean ± SD**	***p****
**Retinal thickness**						
**Central**	274.63 ± 13.50	273.41 ± 16.08	274.90 ± 15.07	270.44 ± 24.12	271.00 ± 16.40	0.441
**Inner inferior**	268.73 ± 12.29	263.92 ± 9.48	269.70 ± 13.37	263.33 ± 13.06	259.44 ± 8.07	0.017#
**Outer inferior**	265.55 ± 11.08	261.33 ± 7.34	264.00 ± 11.46	256.66 ± 8.47	253.55 ± 8.42	0.012#
**Inner superior**	261.64 ± 10.23	255.83 ± 8.41	261.30 ± 16.82	253.44 ± 12.84	247.77 ± 9.53	0.050
**Outer superior**	270.82 ± 10.94	264.08 ± 7.87	267.90 ± 12.10	261.77 ± 13.03	258.55 ± 7.98	0.012#
**Inner nasal**	265.23 ± 11.74	262.33 ± 6.02	262.90 ± 7.95	259.77 ± 9.97	255.33 ± 7.12	0.068
**Outer nasal**	266.23 ± 10.04	262.50 ± 5.36	262.80 ± 7.36	260.66 ± 10.07	254.77 ± 6.64	0.018#
**Inner temporal**	264.95 ± 11.12	258.92 ± 7.65	262.20 ± 11.96	258.22 ± 9.32	252.33 ± 3.60	0.017#
**Outer temporal**	266.95 ± 10.34	260.58 ± 6.33	263.80 ± 12.70	258.25 ± 7.24	255.11 ± 3.44	0.018#
**Total volume**	1.97 ± 0.05	1.97 ± 0.04	1.97 ± 0.05	1.83 ± 0.11	1.81 ± 0.03	0.012#
**RNFL thickness**						
**Global**	46.04 ± 5.65	42.90 ± 3.41	45.90 ± 5.19	43.22 ± 3.30	41.00 ± 3.95	0.058
**Inferior temporal**	45.61 ± 5.08	39.60 ± 6.04	47.10 ± 11.29	41.33 ± 6.00	39.28 ± 5.93	0.075
**Inferior nasal**	45.70 ± 9.10	42.50 ± 5.56	45.80 ± 8.89	43.22 ± 6.75	41.85 ± 7.24	0.237
**Superior temporal**	48.48 ± 11.54	46.90 ± 11.58	47.10 ± 9.25	46.11 ± 10.70	41.42 ± 11.90	0.674
**Superior nasal**	45.42 ± 10.00	44.60 ± 5.54	42.50 ± 5.16	46.11 ± 8.78	42.85 ± 7.10	0.892
**Nasal**	44.09 ± 8.53	39.60 ± 6.60	44.70 ± 5.33	39.66 ± 7.41	37.14 ± 9.40	0.271
**Temporal**	43.35 ± 13.15	45.40 ± 4.45	47.40 ± 10.76	45.11 ± 4.37	43.85 ± 5.36	0.225
**GCL thickness**						
**Central**	20.27 ± 2.54	21.00 ± 2.86	19.80 ± 2.52	18.66 ± 3.24	16.11 ± 2.14	0.020#
**Inner inferior**	28.06 ± 2.23	26.50 ± 3.06	25.80 ± 1.75	22.22 ± 6.88	24.44 ± 2.40	0.027
**Outer inferior**	27.89 ± 1.37	27.17 ± 1.89	26.70 ± 1.33	22.77 ± 7.99	26.00 ± 1.50	0.017#
**Inner superior**	26.50 ± 1.89	24.17 ± 4.56	24.20 ± 3.99	22.22 ± 3.56	20.44 ± 3.43	0.028
**Outer superior**	26.27 ± 2.86	25.67 ± 3.28	26.10 ± 3.57	25.66 ± 1.93	24.33 ± 2.50	0.180
**Inner nasal**	24.68 ± 4.44	23.00 ± 4.30	24.60 ± 2.83	22.33 ± 3.77	20.89 ± 3.33	0.141
**Outer nasal**	25.45 ± 3.55	24.42 ± 3.23	26.40 ± 1.42	24.33 ± 3.77	24.11 ± 3.48	0.799
**Inner temporal**	24.23 ± 4.93	22.50 ± 3.45	22.20 ± 3.19	20.55 ± 5.41	19.78 ± 4.65	0.205
**Outer temporal**	26.50 ± 3.36	24.33 ± 2.70	25.20 ± 2.52	22.75 ± 5.59	22.33 ± 3.57	0.049
**Total volume**	0.18 ± .01	0.17 ± 0.01	0.15 ± 0.01	0.13 ± 0.05	0.15 ± 0.01	0.026

Table S2. Left eye neuroretinal analysis using OCT in both microsphere models at week 8. Ms38/20: 38/20 microsphere model, Ms20/10: 20/10 microsphere model; RNFL: retina nerve fiber layer; GCL: ganglion cell layer complex; thickness in microns (µm); mean ± SD (SD: standard deviation); *: $p < 0.050$ statistical significance; #: $p < 0.020$ statistical significance with Bonferroni correction for multiple comparisons; w: week. Grey cells show the two thinnest sectors at every examination.

OCT parameters (µm)	Left eye					
	(Ms38/20)			(Ms20/10)		
	Baseline Mean ± SD	8 w Mean ± SD	p^*	BASELINE Mean ± SD	8 w Mean ± SD	p^*
**Retinal thickness**						
**Central**	274.81 ± 12.36	262.22 ± 13.35	0.051	279.25 ± 23.01	263.611 ± 32.95	0.050
**Inner inferior**	273.73 ± 8.91	258.22 ± 8.71	0.008#	270.19 ± 9.64	255.61 ± 16.28	0.062
**Outer inferior**	268.64 ± 8.62	253.11 ± 8.97	0.056	265.63 ± 8.60	250.77 ± 13.31	0.058
**Inner superior**	262.36 ± 7.91	248.33 ± 8.60	0.021	261.56 ± 14.01	244.72 ± 24.70	0.050
**Outer superior**	272.82 ± 10.19	259.88 ± 9.70	0.008#	266.37 ± 12.88	256.27 ± 19.34	0.091
**Inner nasal**	266.73 ± 10.57	255.66 ± 10.39	0.038	267.31 ± 11.74	363.94 ± 463.53	0.286
**Outer nasal**	267.64 ± 8.06	256.44 ± 9.68	0.011#	265.56 ± 10.44	252.16 ± 15.05	0.029
**Inner temporal**	268.55 ± 9.49	251.44 ± 9.74	0.008#	269.19 ± 11.64	250.22 ± 21.65	0.050
**Outer temporal**	269.82 ± 8.78	255.22 ± 9.35	0.008#	268.38 ± 9.61	251.22 ± 13.88	0.018#
**Total volume**	1.98 ± 0.02	1.80 ± 0.04	0.007#	1.93 ± 0.07	1.79 ± 0.12	0.050
**RNFL thickness**						
**Global**	46.80 ± 6.10	40.55 ± 9.23	0.123	48.18 ± 6.01	39.94 ± 4.27	0.008#
**Inferior temporal**	45.45 ± 4.86	40.66 ± 11.07	0.172	48.56 ± 9.74	40.88 ± 10.84	0.008#
**Inferior nasal**	46.27 ± 8.61	47.88 ± 16.60	0.161	51.50 ± 10.69	46.27 ± 10.35	0.013#
**Superior temporal**	49.10 ± 9.80	36.33 ± 9.39	0.018#	47.31 ± 13.24	35.33 ± 17.99	0.041
**Superior nasal**	44.60 ± 9.61	38.00 ± 11.34	0.075	43.68 ± 13.36	31.61 ± 16.15	0.050
**Nasal**	44.64 ± 9.59	39.88 ± 12.63	0.235	48.44 ± 7.00	39.72 ± 6.30	0.015#
**Temporal**	45.00 ± 15.89	41.33 ± 11.62	0.475	47.37 ± 14.39	41.44 ± 11.90	0.007#
**GCL thickness**						
**Central**	20.40 ± 2.50	17.22 ± 3.49	0.018#	20.80 ± 3.12	17.44 ± 4.40	0.040
**Inner inferior**	28.78 ± 1.20	25.22 ± 1.56	0.017#	27.08 ± 2.53	24.89 ± 4.54	0.182
**Outer inferior**	28.30 ± 1.25	25.67 ± 1.93	0.020#	26.55 ± 1.69	24.50 ± 3.14	0.042
**Inner superior**	26.90 ± 1.52	21.33 ± 2.23	0.017#	25.47 ± 3.02	20.61 ± 4.88	0.059
**Outer superior**	27.00 ± 2.70	21.67 ± 2.29	0.011#	26.20 ± 2.17	23.83 ± 5.45	0.233
**Inner nasal**	25.20 ± 3.93	21.11 ± 4.34	0.075	21.73 ± 3.34	22.72 ± 5.95	0.837
**Outer nasal**	25.80 ± 3.52	23.22 ± 3.66	0.249	23.20 ± 3.34	23.61 ± 5.19	0.634
**Inner temporal**	24.00 ± 5.20	21.89 ± 4.07	0.496	22.20 ± 4.14	20.17 ± 3.94	0.475
**Outer temporal**	26.40 ± 4.14	23.89 ± 4.07	0.360	24.73 ± 2.65	22.89 ± 4.18	0.183

**Total volume**	0.18 ± 0.01	0.16 ± 0.01	0.027	0.17 ± 0.01	0.15 ± 0.02	0.119

Table S3. Right eye neuroretinal loss rate measured using OCT in the OHT models. EPI: episcleral sclerosis model; Ms38/20: 38/20 microsphere model; Ms20/10: 20/10 microsphere model; RNFL: retina nerve fiber layer; GCL: ganglion cell layer complex; RE: right eye; thickness in microns (µm); w: week. Grey cells show the lowest measurements.

	**RE loss rate (µm)/mmHg/day (All sectors average)**								
**Time**	**RNFL**			**GCL**			**Retina**		
	**EPI**	**Ms38/20**	**Ms20/10**	**EPI**	**Ms38/20**	**Ms20/10**	**EPI**	**Ms38/20**	**Ms20/10**
**2 w**	0.0743	−0.1595	−0.0454	0.0040	−0.0721	−0.0372	−0.0492	−0.2716	−0.0110
**4 w**	0.0438	0.0029	−0.0116	0	−0.0100	−0.0030	−0.0601	−0.0170	−0.0268
**6 w**	−0.0024	−0.0094	−0.0080	−0.0043	−0.0134	−0.0105	−0.0063	−0.0295	−0.0350
**8 w**	−0.0033	−0.0102	−0.0030	−0.009	−0.0072	−0.0059	−0.0095	−0.0221	−0.0145
**Average**	0.0281	−0.0440	−0.017	−0.0018	−0.0257	−0.0142	−0.0313	−0.0851	−0.021

Table S4. Right eye neuroretinal analysis follow-up using OCT in both OHT models. EPI: episcleral sclerosis model; Ms20/10: 20/10 microsphere model; RNFL: retina nerve fiber layer; GCL: ganglion cell layer complex; thickness in microns (µm); mean ± SD (SD: standard deviation); % Ch: percentage change in thickness loss; *: $p < 0.050$ statistical significance; #: $p < 0.020$ statistical significance with Bonferroni correction for multiple comparisons; w: week. Cells colored grey when EPI model showed thinner sectors and/or higher percentage loss compared to Ms20/10.

Right eye structural neuroretinal measurements according to oht models (epivsms20/10)														
**OCT parameters (µm)**	**Baseline**		**2 w**			**4 w**			**6 w**			**8 w**		
	**Mean ± SD**	***p***	**Mean ± SD**	**% Ch**	***p***	**Mean ± SD**	**% Ch**	***p***	**Mean ± SD**	**% Ch**	***p***	**Mean ± SD**	**% Ch**	***p***
**Retinal thickness**														
**Central**	273.80 ± 14.57	0.182	271.66 ± 12.22	−0.78	0.727	271.33 ± 21.38	−0.90	0.827	265.12 ± 19.04	−3.17	0.315	265.91 ± 14.79	−2.88	0.123
	266.69 ± 20.06		268.61 ± 21.20	0.72		265.95 ± 20.00	−0.27		259.76 ± 20.88	−2.59		258.10 ± 14.88	−3.22	
**Inner inferior**	259.90 ± 17.17	0.975	261.66 ± 12.74	0.68	0.896	260.66 ± 4.50	0.29	0.726	261.68 ± 10.22	0.68	0.256	256.16 ± 9.18	−1.44	0.256
	261.46 ± 12.86		262.19 ± 9.99	0.27		260.19 ± 13.66	−0.48		257.66 ± 13.18	−1.45		251.78 ± 11.91	−3.70	
**Outer inferior**	258.30 ± 13.59	0.780	243.33 ± 17.61	−5.80	0.073	244.33 ± 6.11	−5.41	0.080	259.08 ± 13.82	0.30	0.164	246.58 ± 10.57	−4.54	0.612
	258.62 ± 10.18		258.90 ± 8.70	0.10		255.29 ± 12.19	−1.28		252.14 ± 11.11	−2.50		248.68 ± 11.75	−3.84	
**Inner superior**	256.70 ± 12.38	0.291	261.00 ± 7.21	1.68	0.186	244.00 ± 4.35	−4.95	0.485	254.96 ± 14.92	−0.68	0.049	255.00 ± 24.93	−0.66	0.319
	250.77 ± 12.89		252.60 ± 14.21	0.72		250.95 ± 15.77	0.07		246.38 ± 14.81	−1.75		245.72 ± 16.93	−2.01	
**Outer superior**	267.40 ± 15.02	0.263	262.33 ± 6.50	−1.90	0.250	245.00 ± 9.53	−8.38	0.105	262.80 ± 15.22	−1.72	0.049	257.66 ± 18.63	−3.64	0.610
	260.23 ± 9.71		258.32 ± 11.24	−0.73		257.62 ± 13.51	−1.00		254.00 ± 13.93	−2.39		254.52 ± 19.25	−2.19	
**Inner nasal**	258.10 ± 12.99	0.901	261.00 ± 8.18	1.12	0.512	250.00 ± 4.58	−3.14	0.274	255.40 ± 14.03	−1.05	0.310	256.00 ± 14.07	−0.81	0.529
	257.00 ± 10.73		256.71 ± 9.52	−0.11		257.48 ± 13.46	0.18		251.47 ± 12.83	−2.15		251.84 ± 12.02	−2.00	
**Outer nasal**	261.22 ± 12.42	0.462	253.66 ± 2.51	−2.89	0.384	244.33 ± 6.65	−6.47	0.088	257.62 ± 10.87	−1.38	0.103	253.50 ± 15.90	−2.96	0.626
	258.15 ± 8.90		258.10 ± 9.78	−0.01		256.62 ± 13.72	−0.59		251.23 ± 14.09	−2.68		252.41 ± 11.75	−2.22	
**Inner temporal**	261.00 ± 15.84	0.841	259.00 ± 3.60	−0.77	0.999	247.66 ± 4.61	−5.11	0.359	256.00 ± 16.79	−1.92	0.508	256.58 ± 29.11	−1.69	0.792
	259.15 ± 14.12		257.29 ± 11.25	−0.71		254.71 ± 14.14	−1.71		252.09 ± 14.89	−2.72		247.63 ± 13.63	−4.44	
**Outer temporal**	261.80 ± 13.27	0.804	253.00 ± 2.64	−3.36	0.238	243.33 ± 7.50	−7.06	0.088	263.56 ± 19.89	0.67	0.087	258.58 ± 26.94	−1.23	0.570

	291.77 ± 11.56		257.90 ± 10.31	−1.47		255.81 ± 12.46	−2.27		253.95 ± 13.09	−2.98		250.89 ± 14.55	−4.15	
**Total volume**	1.93 ± 0.09	0.418	1.82 ± 0.00	−5.70	0.003#	1.76 ± 0.05	−8.81	0.001#	1.83 ± 0.10	−5.18	0.204	1.81 ± 0.11	−6.22	0.597
	1.88 ± 0.10		1.92 ± 0.23	2.07		1.96 ± 0.07	3.78		1.80 ± 0.09	−4.68		1.74 ± 0.15	−7.86	
**RNFL thickness**														
**Global**	43.44 ± 2.69	0.267	47.66 ± 6.35	9.71	0.179	50.66 ± 7.76	16.64	0.148	42.96 ± 4.42	−1.10	0.311	41.45 ± 3.53	−4.58	0.367
	44.84 ± 3.64		43.70 ± 4.81	−2.55		44.05 ± 6.61	−1.77		43.50 ± 6.47	−3.00		43.44 ± 9.80	−3.13	
**Inferior temporal**	46.20 ± 5.13	0.732	46.00 ± 16.82	−0.43	0.615	42.66 ± 3.21	−7.64	0.631	45.40 ± 10.31	−1.73	0.721	40.36 ± 7.24	−12.64	0.072
	50.54 ± 13.31		51.50 ± 15.47	1.89		47.47 ± 14.95	−6.07		48.11 ± 15.02	−4.80		48.94 ± 16.42	−3.16	
**Inferior nasal**	45.20 ± 7.99	0.400	52.66 ± 5.77	16.50	0.437	48.66 ± 1.52	7.68	0.886	46.96 ± 8.76	3.89	0.786	45.45 ± 5.57	0.55	0.290
	49.15 ± 11.43		48.00 ± 17.56	−2.33		49.00 ± 14.78	−0.30		47.33 ± 11.14	−3.70		49.05	−0.20	
**Superior temporal**	44.89 ± 10.56	0.999	54.66 ± 9.23	21.76	0.027	62.66 ± 14.74	39.61	0.044	38.75 ± 15.03	−13.68	0.469	43.72 ± 11.84	−2.61	0.499
	42.46 ± 8.05		39.37 ± 10.58	−7.27		42.47 ± 10.99	0.02		42.61 ± 12.53	0.35		40.33 ± 13.88	−5.01	
**Superior nasal**	41.44 ± 4.06	0.947	44.66 ± 7.23	7.77	0.201	56.00 ± 20.07	35.14	0.150	33.36 ± 13.67	−19.50	0.107	36.00 ± 14.58	−13.13	0.719
	39.23 ± 13.08		36.75 ± 11.84	−6.32		39.47 ± 15.67	0.60		39.88 ± 15.05	1.65		35.88 ± 17.52	−8.54	
**Nasal**	39.30 ± 6.68	0.320	46.66 ± 5.03	18.73	0.119	43.66 ± 2.30	11.12	0.363	43.76 ± 7.82	11.35	0.004#	41.81 ± 3.40	6.39	0.215
	43.31 ± 9.18		39.35 ± 10.40	−9.14		40.42 ± 9.92	−6.67		38.33 ± 8.87	−11.49		39.94 ± 16.86	−7.78	
**Temporal**	42 ± 10.89	0.709	43.66 ± 8.02	3.98	0.464	53.00 ± 15.71	26.19	0.631	45.32 ± 10.50	7.90	0.482	41.18 ± 7.11	−1.95	0.170
	45.54 ± 6.72		47.20 ± 7.45	3.64		46.42 ± 7.64	1.93		46.44 ± 8.43	1.97		46.94 ± 13.43	3.07	
**GCL thickness**														
**Central**	20.50 ± 2.66	0.321	22.00 ± 3.00	7.32	0.040	20.66 ± 1.52	0.78	0.455	17.76 ± 3.19	−13.37	0.885	18.92 ± 3.47	−7.71	0.009#
	19.16 ± 3.48		18.52 ± 2.46	−3.35		19.61 ± 3.00	2.31		17.71 ± 3.13	−7.60		15.68 ± 2.23	−18.19	
**Inner inferior**	26.50 ± 3.78	0.877	24.00 ± 3.00	−9.43	0.309	25.66 ± 0.57	−3.17	0.560	23.48 ± 4.23	−11.40	0.548	26.25 ± 2.05	−0.94	0.001#
	26.44 ± 3.04		25.71 ± 2.17	−2.76		26.33 ± 1.65	−0.41		24.42 ± 2.87	−7.63		22.21 ± 4.32	−15.99	

**Outer inferior**	27.40 ± 1.67	0.596	26.00 ± 2.64	−5.11	0.627	24.66 ± 2.08	−10.00	0.170	24.48 ± 4.31	−10.66	0.697	26.25 ± 3.59	−4.20	0.276
	26.25 ± 2.25		25.43 ± 2.73	−3.12		26.57 ± 2.18	1.21		25.66 ± 2.30	−2.24		22.74 ± 5.68	−13.37	
**Inner superior**	26.17 ± 2.63	0.570	26.33 ± 2.30	0.61	0.119	23.00 ± 1.73	−12.11	0.508	22.84 ± 3.28	−12.72	0.380	22.42 ± 4.18	−14.33	0.079
	25.25 ± 2.17		22.80 ± 3.59	−9.70		23.80 ± 3.40	−5.74		21.71 ± 3.73	−14.01		20.06 ± 3.88	−20.55	
**Outer superior**	25.50 ± 3.93	0.669	26.33 ± 2.30	3.25	0.524	23.00 ± 3.46	−9.80	0.186	25.04 ± 2.74	−1.80	0.417	23.50 ± 3.89	−7.84	0.754
	26.33 ± 2.27		25.37 ± 1.89	−3.64		25.57 ± 2.27	−2.88		24.80 ± 2.69	−5.81		22.71 ± 5.12	−13.74	
**Inner nasal**	23.83 ± 4.35	0.706	24.00 ± 1.00	0.71	0.235	20.66 ± 4.04	−13.30	0.233	21.88 ± 4.98	−8.18	0.723	23.50 ± 1.78	−1.38	0.034
	23.25 ± 2.83		21.95 ± 3.41	−5.59		22.61 ± 3.35	−2.75		21.71 ± 4.06	−6.62		20.21 ± 4.45	−13.07	
**Outer nasal**	23.67 ± 4.13	0.182	26.33 ± 0.57	11.24	0.129	24.00 ± 3.00	1.39	0.626	23.79 ± 5.25	0.51	0.299	25.50 ± 1.62	7.73	0.140
	25.75 ± 2.05		23.60 ± 3.88	−8.34		24.71 ± 2.55	−4.03		23.19 ± 4.30	−9.94		22.76 ± 5.19	−11.61	
**Inner temporal**	21.67 ± 5.50	0.963	23.33 ± 2.51	7.66	0.271	23.33 ± 2.88	7.66	0.566	21.40 ± 4.76	−1.25	0.207	23.50 ± 4.70	8.44	0.018[#]
	21.83 ± 5.00		21.10 ± 3.41	−3.34		22.71 ± 3.75	4.03		20.00 ± 3.80	−8.38		19.68 ± 3.74	−9.84	
**Outer temporal**	25.17 ± 3.97	0.925	24.66 ± 2.51	−1.99	0.826	24.66 ± 2.88	−2.03	0.628	24.64 ± 4.56	−2.11	0.083	25.42 ± 3.47	0.99	0.147
	24.75 ± 3.74		23.76 ± 3.72	−4.00		24.95 ± 3.33	0.80		23.19 ± 3.85	−6.30		23.26 ± 4.70	−6.02	
**Total volume**	0.17 ± 0.02	0.942	0.17 ± 0.00	0.00	0.363	0.16 ± 0.01	−5.88	0.670	0.13 ± 0.07	−23.53	0.787	0.15 ± 0.04	−11.76	0.076
	0.17 ± 0.02		0.16 ± 0.01	−5.55		0.17 ± 0.01	−1.67		0.15 ± 0.03	−13.24		0.14 ± 0.02	−19.02	
